# COVID-19 in children in the state of Pernambuco: Spatial analysis of confirmed severe cases and the Human Development Index

**DOI:** 10.1590/0037-8682-0782-2020

**Published:** 2021-03-22

**Authors:** Amanda Priscila de Santana Cabral Silva, Eliane Rolim de Holanda, Paula Daniella de Abreu, Marcelo Victor de Arruda Freitas

**Affiliations:** 1 Universidade Federal de Pernambuco, Centro Acadêmico Vitória, Núcleo de Saúde Coletiva, Vitória de Santo Antão, PE, Brasil.; 2 Fundação Oswaldo Cruz, Instituto Aggeu Magalhães, Departamento de Saúde Coletiva, Recife, PE, Brasil.; 3 Universidade Federal de Pernambuco, Centro Acadêmico Vitória, Núcleo de Enfermagem, Vitória de Santo Antão, PE, Brasil.; 4 Universidade de São Paulo, Escola de Enfermagem de Ribeirão Preto, Programa de Pós-Graduação de Enfermagem em Saúde Pública, Ribeirão Preto, SP, Brasil.

**Keywords:** Pandemic, Coronavirus infections, Child health, Spatial analysis, Surveillance, Public health

## Abstract

**INTRODUCTION:**

Health planning is required for the control and prevention of severe cases of COVID-19 in children.

**METHODS:**

Spatial analysis of severe COVID-19 cases in children of Pernambuco in the first six months of the pandemic and its autocorrelation with the Human Development Index was conducted.

**RESULTS:**

A total of 551 severe cases (39.4 cases/100,000 inhabitants) was initially concentrated in the metropolitan area, with later interiorization. The spatial autocorrelation of cases was identified. The bivariate analysis revealed alert regions in less developed municipalities (I=0.341; p=0.001).

**CONCLUSIONS:**

Considering the local particularities can assist in directing the priorities for decision making.

Coronavirus disease 2019 (COVID-19), a disease caused by the severe acute respiratory syndrome coronavirus 2 (SARS-CoV-2), was first recorded in December 2019 in the city of Wuhan, Province of Hubei, China. It was declared a public health emergency of international interest in January 2020 and a pandemic in March of the same year^1^.

Initially, COVID-19 was described as a disease that occurred predominantly in adults and was associated with pneumonia of unknown etiology^2^. The pandemic spread as a result of the high rates of contamination. Pediatric cases appeared less frequently, with a lower frequency of hospitalizations, complications, and fatal outcomes, compared to adults and the elderly^3^. The most severe cases were associated with profiles of children with pre-existing comorbidities^3^.

Despite the collective efforts to contain the pandemic, social inequalities in Brazil are fertile ground for spreading COVID-19. Social isolation is difficult. There is restricted access to basic supplies for hygiene and protection and also limited access to health services. The disparities between the number of beds and respirators per capita in the public and private sectors, and between capitals and hinterland cities, generate distortions that disrupt the efficient distribution of resources and contribute to mortality^4^.

Severe COVID-19 cases in children represent the most detrimental manifestations of the disease in this age group, that places greater demands om the health system. This study aimed to analyze the spatial distribution of severe COVID-19 cases in children and its correlation with the Municipal Human Development Index in the state of Pernambuco.

This ecological study was performed in Pernambuco. The state is divided into 185 municipalities distributed over12 health regions that are grouped into four macro-health regions (Metropolitan, Agreste, Sertão, and Vale do São Francisco and Araripe) (Figure 1). In 2019, the population was estimated to be 9,557,071 inhabitants, of which 15.0% (n=1,397,623) were under 10 years old^5^.

**FIGURE 1:**
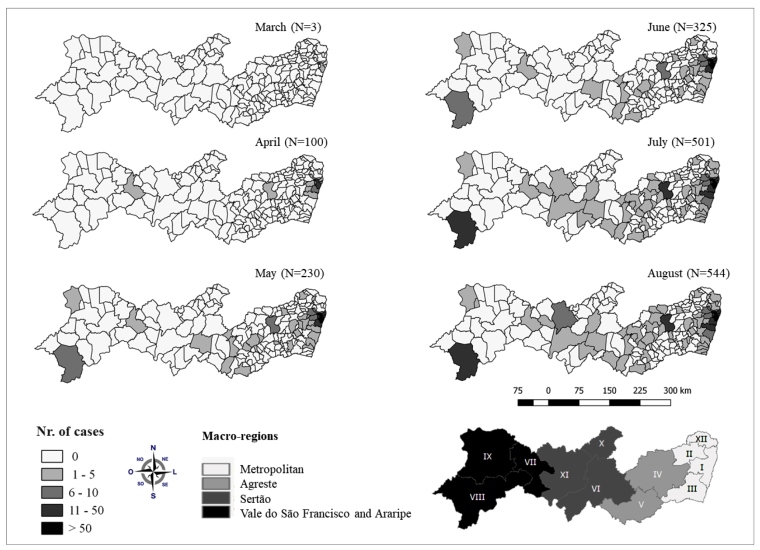
Monthly evolution of the spatial distribution of severe cases of COVID-19 in children under 10 years old by residential municipality. Pernambuco, March to August 2020*. Source: Own elaboration * Epidemiological weeks 10 to 35 / 2020.

The study population included individuals under 10 years of age with confirmed severe COVID-19, notified between March 1 and August 29, 2020, which corresponded to epidemiological weeks (Epi week) 10 to 35 of this year. For notification purposes, a case was considered “severe” if the individual was hospitalized for the severe acute respiratory syndrome (SARS), defined as a flu-like syndrome, presenting with dyspnea or persistent pressure in the chest, or O_2_ saturation below 95% in room air, or signs of respiratory discomfort.

The rates of detection of severe COVID-19 in children were calculated for each municipality. Local empirical Bayes smoothing was applied to smooth the estimates of coefficients calculated for the small (or underreported) geographical areas, thereby eliminating random fluctuations not associated with the actual risk^6^.

From the smoothed rates, the existence of univariate and bivariate spatial autocorrelation was investigated using the Global Moran’s Index. This index provides a single value ranging from -1 to 1, with 0 being the absence of spatial correlation, a positive value indicating direct correlation, and a negative value indicating inverse correlation. The location of clusters among the classifications in the univariate analysis was detected through local Moran’s analysis as follows:

Not significant;

- High-High (positive values, positive means) and Low-Low (negative values, negative means): indicate a positive spatial association, municipalities where neighbors have similar values;
- High-Low (positive values, negative means) and Low-High (negative values, positive means): indicate a negative spatial association, municipalities where neighbors have different values.

In the bivariate analysis, the smoothed detection rate of severe COVID-19 in children was considered as a dependent variable. The independent variable was the Municipal Human Development Index obtained by the geometric mean of indices of income, education, and longevity dimensions^7^. These are basic and universal dimensions of life, which influence the choices and opportunities of individuals^8^. In this correlation analysis, the clusters were classified as described by Maciel et al. (2020)^9^:

Not significant;

- High-High: regions formed by municipalities with high frequencies of the dependent variable and high frequencies of the independent variable;
- Low-Low: regions formed by municipalities with low frequencies of the dependent variable and low frequencies of the independent variable;
- High-Low: regions formed by municipalities with high frequencies of the dependent variable and low frequencies of the independent variable;
- Low-High: regions formed by municipalities with low frequencies of the dependent variable and high frequencies of the independent variable.

Clusters that fit in the High-High quadrant of the Moran Map were defined as critical areas and those in the High-Low quadrant were defined as alert areas.

Additionally, the Kappa coefficient was used to categorize the degree of univariate and bivariate spatial autocorrelation generated by the Global Moran’s Index of detection rates of severe COVID-19 cases in children. Values were interpreted as insignificant (<0.0), reasonable (0.0-0.2), weak (0.21-0.4), moderate (0.41-0.6), strong (0.61-0.8), and almost perfect (0.81-1.0).

Spatial analyses were performed using electronic spreadsheets and GeoDA Version 1.14.0 and the QGis 2.18.9 software. The results were represented in the cartographic base of Pernambuco provided by the Brazilian Institute of Geography and Statistics (IBGE)^10^.

Data from cases (obtained on September 4, 2020) are made available by the State Health Department on the “COVID data” (*Covid em dados*) platform managed by the Pernambuco State Planning Department^11^. The official population estimate for each municipality was obtained from the Brazilian Institute of Geography and Statistics^5^. The Human Development Index was captured in the Human Development Atlas in Brazil^7^. In this study, we used a secondary database, publicly available, with no possibility of identifying individuals. Therefore, approval by an ethics committee was deemed unnecessary.

In Pernambuco, between Epi weeks 10 and 35 of 2020, 551 severe COVID-19 cases in children were confirmed, representing 39.4 cases/100 thousand inhabitants. More than half of the cases (n=288) were concentrated specifically between Epi week 23 and Epi week 31, which corresponded to the months of June and July 2020, respectively.

Since the record of residential municipality was available in 544 out of the total number of cases, it was possible to perform the spatial analysis. The monthly evolution of the distribution of severe cases in the state showed the initial concentration of cases in municipalities in the metropolitan region of Recife, with subsequent interiorization, especially from May 2020 (Figure 1). In August 2020, 101 municipalities confirmed at least one severe case of COVID-19 in children under the age of 10 years (Figure 1).

Twenty municipalities concentrated mostly in the metropolitan macro-region had a higher detection rate than the state average, above 40.0 cases/100 thousand inhabitants (Figure 2). Considering the number of cases per 100 thousand inhabitants, the municipalities of Recife (87.6), Camaragibe (69.5), Olinda (68.9), and São Lourenço da Mata (62.8) stood out.

**FIGURE 2:**
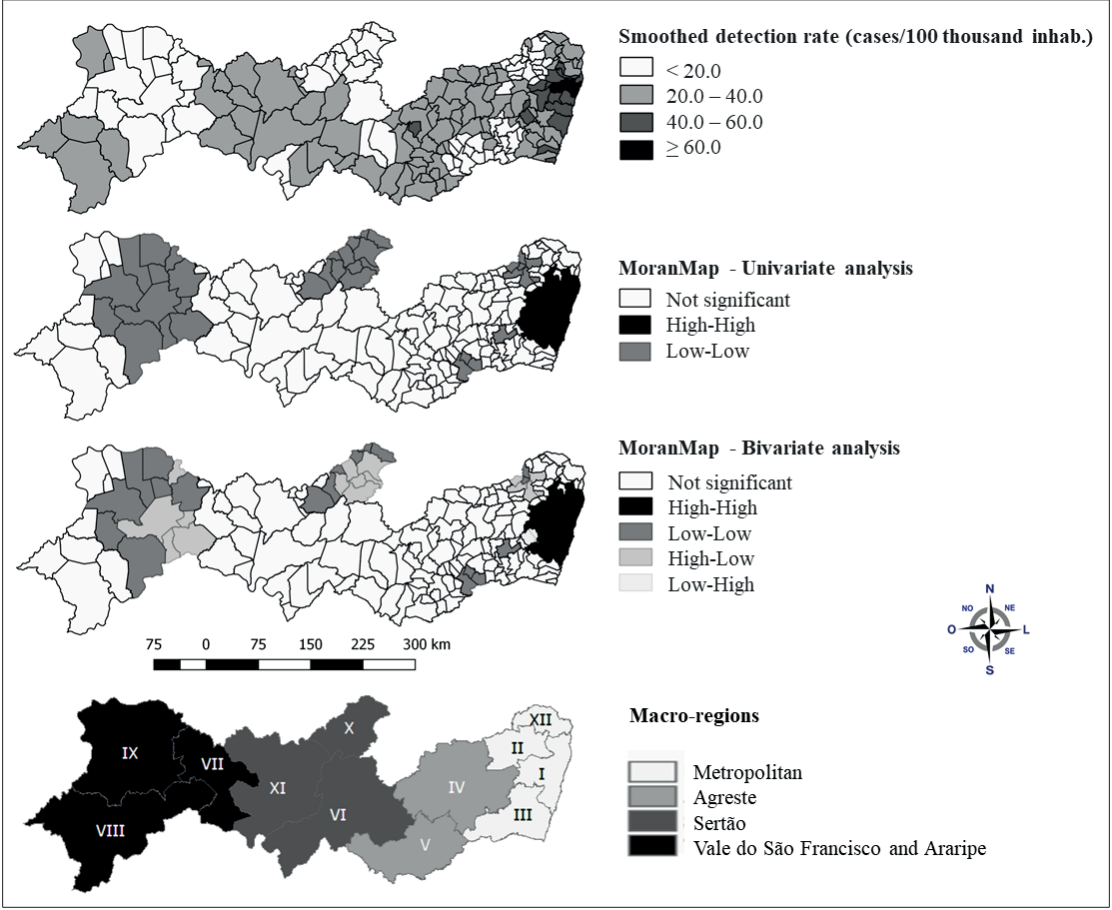
Smoothed detection rate of severe cases of COVID-19 in children under 10 years old and distribution by univariate and bivariate analyzes. Pernambuco, March to August 2020. Source: Own elaboration. *Epidemiological weeks 10 to 35 / 2020.

Univariate analysis identified the presence of a positive and strong spatial autocorrelation between the municipal detection rates (I=0.73; p=0.001). The local Moran analysis revealed a critical region (High-High) formed by municipalities belonging to the metropolitan and agreste macro-regions (Figure 2). In the univariate analysis, in addition to the High-High cluster, there was also a Low-Low pattern distributed in the four macro-regions of the state (Figure 2).

In the Moran Global bivariate analysis between the detection rates of severe cases of COVID-19 in children and the Municipal Human Development Index, a significant positive correlation was found (I=0.341; p=0.001). When inserting the Municipal Human Development Index as the independent variable, the High-High cluster remained. In particular, in the areas classified as Low-Low in the previous analysis, alert regions were revealed (Figure 2). In this scenario, we highlighted the significant municipalities for the High-Low spatial pattern located in the following macro-regions: Metropolitan (Bom Jardim, Lagoa do Carro, Limoeiro, Orobó and Vicência), Sertão (Afogados da Ingazeira, Ingazeira, Iguaraci, São José do Egito, Tabira, and Tuparetama), and Vale do São Francisco and Araripe (Cabrobó, Moreilandia, Orocó, Parnamirim, and Terra Nova).

Regarding human development, the presence of moderate positive spatial autocorrelation was identified in the distribution of the MHDI in Pernambuco (I=0.46; p=0.001), as detailed in Table 1.

**TABLE 1:** Distribution of the Municipal Human Development Index (MHDI), Moran Index and p-value. Pernambuco, Brazil 2020.

Municipalities	MHDI	Moran Index	p-value
Água Preta	0.553	0.097	0.398
Águas Belas	0.526	1.918	0.000
Abreu e Lima	0.679	2.094	0.007
Afogados da Ingazeira	0.657	-0.074	0.474
Afrânio	0.588	-0.167	0.065
Agrestina	0.592	-0.011	0.350
Alagoinha	0.599	-0.010	0.440
Aliança	0.604	0.068	0.187
Altinho	0.598	0.003	0.423
Amaraji	0.580	-0.010	0.431
Angelim	0.572	0.395	0.032
Araçoiaba	0.592	-0.081	0.016
Araripina	0.602	-0.076	0.184
Arcoverde	0.667	-0.557	0.236
Barra de Guabiraba	0.577	0.073	0.372
Barreiros	0.586	0.047	0.370
Belém de Maria	0.578	0.211	0.100
Belém de São Francisco	0.642	0.450	0.190
Belo Jardim	0.629	-0.416	0.048
Betânia	0.559	-0.113	0.370
Bezerros	0.606	-0.006	0.455
Bodocó	0.565	0.247	0.213
Bom Conselho	0.563	0.852	0.002
Bom Jardim	0.602	0.009	0.421
Bonito	0.561	0.257	0.142
Brejão	0.547	0.491	0.186
Brejinho	0.574	-0.118	0.288
Brejo da Madre de Deus	0.562	-0.239	0.203
Buíque	0.527	0.935	0.047
Buenos Aires	0.593	-0.051	0.009
Cabo de Santo Agostinho	0.686	2.507	0.004
Cabrobó	0.623	0.386	0.086
Cachoeirinha	0.579	0.012	0.449
Caetés	0.522	0.477	0.245
Calçados	0.566	0.290	0.189
Calumbi	0.571	-0.193	0.189
Camaragibe	0.692	4.885	0.000
Camocim de São Félix	0.588	0.080	0.130
Camutanga	0.606	0.051	0.332
Canhotinho	0.541	0.856	0.017
Capoeiras	0.549	0.151	0.336
Carnaíba	0.583	0.004	0.506
Carnaubeira da Penha	0.573	-0.383	0.046
Carpina	0.680	1.217	0.050
Caruaru	0.677	-0.064	0.476
Casinhas	0.567	-0.100	0.372
Catende	0.609	-0.185	0.031
Cedro	0.615	0.354	0.137
Chã de Alegria	0.604	0.166	0.055
Chã Grande	0.599	0.005	0.449
Condado	0.602	0.067	0.172
Correntes	0.536	0.457	0.256
Cortês	0.568	0.139	0.275
Cumaru	0.572	0.088	0.342
Cupira	0.592	0.038	0.087
Custódia	0.594	0.007	0.247
Dormentes	0.589	0.007	0.474
Escada	0.632	0.455	0.073
Exu	0.576	0.080	0.383
Feira Nova	0.600	0.044	0.178
Ferreiros	0.622	0.072	0.423
Flores	0.556	0.055	0.462
Floresta	0.626	-0.162	0.243
Frei Miguelinho	0.576	-0.067	0.342
Gameleira	0.602	-0.016	0.433
Garanhuns	0.664	-1.698	0.000
Glória do Goitá	0.604	0.066	0.157
Goiana	0.651	0.645	0.085
Granito	0.595	0.001	0.349
Gravatá	0.634	-0.134	0.306
Iati	0.528	1.355	0.025
Ibimirim	0.552	0.884	0.004
Ibirajuba	0.580	0.202	0.057
Igarassu	0.665	1.567	0.002
Iguaraci	0.598	0.025	0.140
Ilha de Itamaracá	0.653	1.530	0.024
Inajá	0.523	1.254	0.035
Ingazeira	0.608	0.142	0.174
Ipojuca	0.619	0.499	0.059
Ipubi	0.550	0.257	0.319
Itaíba	0.510	3.025	0.000
Itacuruba	0.595	-0.002	0.217
Itambé	0.575	-0.214	0.129
Itapetim	0.592	-0.014	0.362
Itapissuma	0.633	1.123	0.018
Itaquitinga	0.586	-0.187	0.020
Jaboatão dos Guararapes	0.717	5.658	0.000
Jaqueira	0.575	0.385	0.037
Jataúba	0.530	-0.006	0.499
Jatobá	0.645	0.068	0.402
Joaquim Nabuco	0.554	0.281	0.231
João Alfredo	0.576	-0.124	0.265
Jucati	0.550	0.110	0.418
Jupi	0.575	0.179	0.133
Jurema	0.509	1.455	0.041
Lagoa de Itaenga	0.602	0.123	0.029
Lagoa do Carro	0.609	0.275	0.039
Lagoa do Ouro	0.525	0.604	0.206
Lagoa dos Gatos	0.551	0.424	0.137
Lagoa Grande	0.597	0.010	0.307
Lajedo	0.611	-0.176	0.118
Limoeiro	0.663	0.135	0.376
Macaparana	0.609	-0.030	0.456
Machados	0.578	0.031	0.455
Manari	0.487	3.518	0.002
Maraial	0.534	0.524	0.180
Mirandiba	0.591	-0.059	0.069
Moreilândia	0.600	-0.015	0.457
Moreno	0.652	2.176	0.001
Nazaré da Mata	0.662	0.504	0.159
Olinda	0.735	10.657	0.000
Orobó	0.610	-0.153	0.161
Orocó	0.610	0.063	0.323
Ouricuri	0.572	0.280	0.065
Palmares	0.622	-0.452	0.020
Palmeirina	0.549	0.418	0.155
Panelas	0.569	0.463	0.011
Paranatama	0.537	0.487	0.214
Parnamirim	0.599	-0.012	0.350
Passira	0.592	-0.015	0.236
Paudalho	0.639	1.045	0.001
Paulista	0.732	6.770	0.000
Pedra	0.567	0.391	0.015
Pesqueira	0.610	-0.068	0.229
Petrolândia	0.623	0.263	0.214
Petrolina	0.697	-0.192	0.479
Poção	0.528	0.181	0.437
Pombos	0.598	0.018	0.250
Primavera	0.580	-0.098	0.201
Quipapá	0.552	1.218	0.001
Quixaba	0.577	0.228	0.209
Recife	0.772	9.507	0.000
Riacho das Almas	0.570	-0.221	0.177
Ribeirão	0.602	-0.029	0.276
Rio Formoso	0.613	-0.050	0.392
Sairé	0.585	0.010	0.498
Salgadinho	0.534	-0.369	0.244
Salgueiro	0.669	0.365	0.246
Saloá	0.559	0.595	0.019
Sanharó	0.603	0.059	0.270
Santa Cruz	0.549	0.342	0.220
Santa Cruz da Baixa Verde	0.612	0.317	0.080
Santa Cruz do Capibaribe	0.648	-0.450	0.242
Santa Filomena	0.533	0.762	0.144
Santa Maria da Boa Vista	0.590	0.016	0.437
Santa Maria do Cambucá	0.548	0.142	0.431
Santa Terezinha	0.593	-0.010	0.318
São Benedito do Sul	0.530	1.237	0.018
São Bento do Una	0.593	0.012	0.236
São Caetano	0.591	0.002	0.486
São Joaquim do Monte	0.537	0.256	0.323
São João	0.570	0.162	0.268
São José da Coroa Grande	0.608	-0.057	0.416
São José do Belmonte	0.610	0.171	0.162
São José do Egito	0.635	0.112	0.343
São Lourenço da Mata	0.653	2.206	0.000
São Vicente Ferrer	0.549	-0.126	0.345
Serra Talhada	0.661	-0.112	0.416
Serrita	0.595	-0.002	0.176
Sertânia	0.613	-0.165	0.156
Sirinhaém	0.597	0.014	0.191
Solidão	0.585	-0.098	0.217
Surubim	0.635	-0.488	0.031
Tabira	0.605	0.083	0.172
Tacaimbó	0.554	0.088	0.441
Tacaratu	0.573	-0.098	0.331
Tamandaré	0.593	0.005	0.437
Taquaritinga do Norte	0.641	0.494	0.125
Terezinha	0.545	0.292	0.294
Terra Nova	0.599	0.048	0.142
Timbaúba	0.618	0.155	0.245
Toritama	0.618	0.423	0.076
Tracunhaém	0.605	0.229	0.004
Trindade	0.595	0.002	0.208
Triunfo	0.670	-0.565	0.286
Tupanatinga	0.519	1.656	0.013
Tuparetama	0.634	0.348	0.218
Venturosa	0.592	0.040	0.087
Verdejante	0.605	0.134	0.148
Vertente do Lério	0.563	0.186	0.333
Vertentes	0.582	-0.108	0.197
Vicência	0.605	0.064	0.200
Vitória de Santo Antão	0.640	0.673	0.032
Xexéu	0.552	0.330	0.225

Source: Authors’ elaboration based on the Human Development Atlas in Brazil^7^.

In this study, within the first six months of the pandemic in Pernambuco, the spatial autocorrelation of the occurrence of severe cases of COVID-19 in the pediatric population and the statistically significant cluster with a High-High pattern was located between the Metropolitan and Agreste Macro-regions. The bivariate analysis revealed alert regions (High-Low pattern), which suggested that the living conditions influenced the active spread of the virus among children in the state. Moreover, the effects of the pandemic on this age group may be even more prominent in the less developed areas.

This scenario reinforced that the pandemic course involved not only the transmission and susceptibility characteristics of COVID-19 but also different severities and impairments resulting from social inequality, precarious living, work, income, housing and sanitation conditions, low educational level, and difficulty in accessing water and health services, especially for families residing in urban agglomerations or rural areas far from health facilities and with limited transport infrastructures. These intense social vulnerabilities result in health disparities, as shown in the maps.

Regarding social distance, children felt strong direct and indirect effects on their physical and psychological health^12^. The availability of diagnostic tests was also limited for children, since they were offered to more unwell patients, thereby leading to a probable pediatric underreporting^13^.

These factors added together revealed the lack of effective social protection policies and limitations to guarantee families’ access to emergency aid from the federal government. The lack of incentives in the face of the pandemic is reflected in the insufficient primary health care actions and the lack of social protection.

The new severe COVID_19 cases in Pernambuco showed similar characteristics in the clusters, which indicated a direct association between the cases studied and the Municipal Human Development Index. Although this association is only weak because of the locoregional heterogeneity. Social inequality is evident in the territorial areas of this state, which implies the diversity of factors inherent to specific social dynamics.

From the data of this study, it can be seen that at the beginning of the pandemic, severe COVID-19 cases in children were more concentrated in the municipal urban centers. Over the months, dissemination progressed rapidly to the countryside. Brazil is a country with vast territorial extensions, and the economic, social, and cultural inequalities are among the greatest challenges in ensuring equitable actions in health and healthcare.

During the COVID-19 pandemic, there was an increase in severe cases of COVID-19 in the population under 10 years of age. The available figures may be associated with possible underreporting. This finding serves as a public health warning. Although children are less susceptible to severe forms of COVID-19, the involvement of this age group may be indicative of a family contagion and the possibility of prolonged dissemination by the child/children, even after recovery from their symptomatic condition^13^.

In addition to family contacts, another contagion factor is the history of exposure to epidemic areas or both. In a study conducted with 171 children infected by SARS-CoV-2 admitted to the Children’s Hospital in Wuhan, 90.1% had contact with infected family members or with suspected COVID-19 cases^14^.

Children are susceptible to SARS-CoV-2 and can display asymptomatic or mild forms of COVID-19. Transmissibility in children has contributed substantially to the chain of community transmission, with considerable viral dissemination^15^. From this perspective, attention should not be focused only on the disease, but on the severity of dissemination and daily life in which the health-disease process is evidenced by spatially determining the social relations that must be considered in public health policies.

The underreporting of COVID-19 cases in children due to the scarcity of tests, testing only symptomatic individuals, and the use of secondary data subject to constant variations, were the limitations of this study. However, these data have been widely used as a consistent tool to support management and epidemiological investigations.

Finally, the distribution of pediatric COVID-19 did not occur homogeneously among the municipalities in Pernambuco. The high rates correlated with the areas where children live. The progression of severe infection among children in the less developed municipalities should serve as a warning to health policy makers to “geo-target” human/financial resources and control and surveillance measures for vulnerable communities. In addition, the municipalities with an absence of severe COVID-19 in children or those with Low-Low patterns still need combined measures to prevent the disease from spreading to these locations and thus, should be monitored.
